# Higher Incidence of Stroke in Severe COVID-19 Is Not Associated With a Higher Burden of Arrhythmias: Comparison With Other Types of Severe Pneumonia

**DOI:** 10.3389/fcvm.2021.763827

**Published:** 2021-11-24

**Authors:** Peter Jirak, Zornitsa Shomanova, Robert Larbig, Daniel Dankl, Nino Frank, Clemens Seelmaier, Dominyka Butkiene, Michael Lichtenauer, Moritz Mirna, Bernhard Strohmer, Jan Sackarnd, Uta C. Hoppe, Jürgen Sindermann, Holger Reinecke, Gerrit Frommeyer, Lukas J. Motloch, Rudin Pistulli

**Affiliations:** ^1^Clinic II for Internal Medicine, University Hospital Salzburg, Paracelsus Medical University, Salzburg, Austria; ^2^Department of Cardiology I—Coronary and Peripheral Vascular Disease, Heart Failure, University Hospital Münster, Münster, Germany; ^3^Department of Cardiology II—Electrophysiology, University Hospital Münster, Münster, Germany; ^4^Division of Cardiology, Hospital Maria Hilf Mönchengladbach, Mönchengladbach, Germany; ^5^Department of Anesthesiology, Perioperative Care, and Intensive Care Medicine, University Hospital Salzburg, Paracelsurs Medical University, Salzburg, Austria

**Keywords:** COVID-19, arrhythmias, atrial fibrillation, stroke, pneumonia, ventricular tachycardia, anticoagulation

## Abstract

**Aims:** Thromboembolic events, including stroke, are typical complications of COVID-19. Whether arrhythmias, frequently described in severe COVID-19, are disease-specific and thus promote strokes is unclear. We investigated the occurrence of arrhythmias and stroke during rhythm monitoring in critically ill patients with COVID-19, compared with severe pneumonia of other origins.

**Methods and Results:** This retrospective study included 120 critically ill patients requiring mechanical ventilation in three European tertiary hospitals, including *n* =60 COVID-19, matched according to risk factors for the occurrence of arrhythmias in *n* = 60 patients from a retrospective consecutive cohort of severe pneumonia of other origins. Arrhythmias, mainly atrial fibrillation (AF), were frequent in COVID-19. However, when compared with non-COVID-19, no difference was observed with respect to ventricular tachycardias (VT) and relevant bradyarrhythmias (VT 10.0 vs. 8.4 %, *p* = *ns* and asystole 5.0 vs. 3.3%, *p* = *ns*) with consequent similar rates of cardiopulmonary resuscitation (6.7 vs. 10.0%, *p* = *ns*). AF was even more common in non-COVID-19 (AF 18.3 vs. 43.3%, *p* = *0.003*; newly onset AF 10.0 vs. 30.0%, *p* = *0.006*), which resulted in a higher need for electrical cardioversion (6.7 vs. 20.0%, *p* = *0.029*). Despite these findings and comparable rates of therapeutic anticoagulation (TAC), the incidence of stroke was higher in COVID-19 (6.7.% vs. 0.0, *p* = *0.042*). These events also happened in the absence of AF (50%) and with TAC (50%).

**Conclusions:** Arrhythmias were common in severe COVID-19, consisting mainly of AF, yet less frequent than in matched pneumonia of other origins. A contrasting higher incidence of stroke independent of arrhythmias also observed with TAC, seems to be an arrhythmia-unrelated disease-specific feature of COVID-19.

## Introduction

The novel coronavirus disease COVID-19 caused by severe acute respiratory syndrome coronavirus 2 (SARS-CoV-2) has caused a worldwide healthcare crisis with an overstrain of hospital resources (1, 2). Given its diverse cardiovascular involvement, further investigation of potential disease-specific processes is crucial to optimize its medical management (3–5). Although previous studies observed a high rate of cardiac injury in COVID-19 infections (3), two recent publications reported rates of cardiac injury to be similar to non-COVID-19 pneumonia, pointing against a COVID-19 specific cardiac involvement (6, 7). Similarly, the impact of COVID-19 on cardiac arrhythmias and thromboembolic events is also yet to be covered to the full extent. The arrhythmic burden is high in COVID-19 patients. The first investigation from Wang et al. reported cardiac arrhythmias in 17% of all their included patients and in 44.4% of those admitted to ICU (8). However, the missing definition of arrhythmias in that study should be taken into account when interpreting results (8). A recent work of Bertini et al. analyzed ECGs in critically ill COVID-19 patients and reported a high rate of ECG abnormalities (93%) with atrial fibrillation/flutter being the most common arrhythmia (22%) (9). In this context, the high incidence of stroke in COVID-19, as the most frequent thromboembolic complication of atrial fibrillation, attracts special attention (10–12). Similar investigations on thromboembolic events including stroke, deep vein thrombosis (DVT), and pulmonary embolism reported overall rates of up to 43% in critically ill COVID-19 patients (13–15). Of note, the majority of patients in those studies received at least a prophylactic anticoagulation (13–15). These findings suggest a potential correlation between cardiac arrhythmias and high rates of stroke and other thromboembolic events. Moreover, it remains unclear, whether the high arrhythmic burden in COVID 19 is the effect of unspecific proarrhythmogenic states promoted by cardiac injury as well as the systemic inflammatory burden, or whether a COVID-19 specific mechanism exists, which promotes cardiac arrhythmias. Given its considerable clinical impact, further investigation on COVID-19 associated arrhythmias and their potential link to thromboembolic events is urgently needed. Accordingly, in our multicentre study, we aimed for a comparative analysis of cardiac arrhythmias as well as stroke and other thromboembolic events in critically ill patients requiring ventilator therapy due to SARS-CoV-2 induced pneumonia matched to a historical cohort requiring respiratory support due to severe pneumonia of non-COVID-19 origin (non-COVID-19).

## Methods

The present retrospective study was conducted in three European tertiary centers in Germany and Austria (University Hospital Münster, Maria Hilf Hospital Mönchengladbach and the University Hospital Salzburg). The study was conducted in accordance with the Declaration of Helsinki and the standards of good clinical practice. All three local ethic committees approved the present study (University Hospital Münster Nr. 2020-306-f-S, Maria Hilf Hospital Mönchengladbach: Nr. 143/2020, and University Hospital Salzburg: Nr. 1071/2020).

### Study Cohorts

A total of 120 patients were involved in this study (60 COVID-19 vs. 60 non-COVID-19). The COVID-19 cohort consisted of 60 consecutive patients with available ICU rhythm monitoring who suffered severe pneumonia. Severe pneumonia was defined as pneumonia-associated respiratory failure requiring mechanical ventilation [noninvasive ventilation (NIV) or invasive ventilation]; the term NIV in this study refers to mechanical ventilation involving end-expiratory and inspiratory positive air pressure support *via* a tightly fitted face mask or helmet, as opposed to invasive ventilation necessitating endotracheal intubation. All patients included in the study had some form of mechanical ventilation (patients who merely needed oxygen insufflations were not included) between March and May 2020. Patients were treated according to recent recommendations (1). All patients received anticoagulation during their ICU stay. A detailed description with regards to anticoagulation is given in the Supplementary Methods section. Patients with a history of hyperthyroid disease, of inherited arrhythmic disorders, and a history of persistent or permanent atrial fibrillation (AF) were excluded from the analyses. The diagnosis of COVID-19 was established in the presence of a positive result in real-time reverse transcription–polymerase chain reaction assay (performed according to the manufacturer) for COVID-19 and a chest radiography and/or computer tomography of the thorax indicative for COVID-19 related pneumonia according to current recommendations (2).

The control group was recruited from a consecutive collective of 1,222 patients suffering severe pneumonia of non-COVID-19 origin. All patients in the control group were treated between January 2014 and Mach 2020 at the ICU according to current intensive care guidelines (3). Patients from the control group requiring mechanical ventilation (non-invasive/invasive ventilation) were primarily matched to the COVID-19 population according to the medical history of paroxysmal AF. To account for potential confounders as proarrhythmic comorbidities, patients were further matched for known risk factors associated with cardiac arrhythmias. Matching was conducted stepwise and manually according to age, gender, heart failure, coronary artery disease, atrial flutter, diabetes mellitus, arterial hypertension, valvular heart disease, and previous stroke/TIA. If more than one candidate in the retrospective non-COVID-19 cohort fully fulfilled the matching criteria, the patient with the closest admission time point as compared with the time point of the beginning of the recruitment of the COVID-19 cohort (March 2020) was chosen for matching. To further validate the matching process, covariate imbalance was assessed. Standardized differences and omnibus test revealed no statistically significant covariate imbalance between the two investigated groups (Supplementary Table 6).

### Data Collection and Analyses

In all eligible patients, data were retrospectively collected from electronic medical records. Data obtained comprises demographics, medical history, laboratory examinations, comorbidities, complications, specific treatment measures, and outcomes, and also 12-lead ECGs at ICU admission and complete rhythm monitoring during ICU stay (continuous standard three-lead ECG during complete ICU stay). Laboratory samples were collected within the first hours after ICU admittance, and follow-up was conducted on a daily routine according to the need for clinical assessment. With regards to rhythm monitoring, baseline rhythm was evaluated and documented every hour during the entire ICU stay. Analyses of ECGs, classification of arrhythmias, and quantification of the duration of arrhythmias in the rhythm monitoring were analyzed and documented by a trained team of ICU nurses and physicians in one of the recruiting centers. Cardiac arrhythmias during ICU rhythm monitoring were classified according to current guidelines (4–6). AF was defined as the presence of an irregular rhythm with fibrillatory waves and no defined P-waves for at least 30 s during rhythm monitoring. Other SVTs were defined as regular rhythm when atrial and/or ventricular rates exceeded 100 bpm for at least 30 s during monitoring, consistent with atrial flutter, focal atrial tachycardia, atrioventricular nodal tachycardia, or atrioventricular tachycardia. Non-sustained ventricular tachycardia was defined as three or more consecutive ventricular beats occurring at a rate of ≥100 bpm and sustained ventricular tachycardia lasting ≥30 s. High grade atrioventricular block was defined as the presence of second- or third-degree heart block. Bradyarrhythmia absoluta was defined as the presence of an irregular rhythm with fibrillatory waves and no defined P-waves as well as heart rate <40/min for at least 30 s. Asystole was defined as the absence of electrical activity during rhythm monitoring lasting >6 s. New-onset AF was defined as AF during ICU monitoring in the absence of AF history, as indicated by the medical record of the patient.

Diagnosis of thromboembolic/thrombotic events including pulmonary embolism, thromboembolic stroke, and transient ischemic attack was established in agreement with current guidelines (7, 8). The diagnosis of thromboembolic stroke and transient ischemic attack of thromboembolic origin was verified by an experienced neurologist. Acquired data were independently reviewed and entered into the computer database by two blinded analysts. During ICU stay all recruited patients received standard prophylactic anticoagulation or therapeutic anticoagulation (TAC), if indicated, using low molecular weight heparin.

### Statistical Analysis

Statistical analysis was conducted using R (version 4.0.2., R Core Team (2013), R Foundation for Statistical Computing, Vienna, Austria; http://www.R-project.org/) using the packages “MatchIt,” “optmatch” and “RItools,” “stddiff,” and also SPSS (Version 23.0, IBM, Armonk, New York, USA), and was carried out blindly by our statistical analytic team. Descriptive statistics were obtained for all study variables. All categorical variables were compared by using the Fisher exact test. Ordinal data are presented as median (interquartile range [IQR]). Median values were compared using the Mann–Whitney-U test. Normal distribution of continuous variables was tested using the Kolmogorov–Smirnov test. According to results, continuous variables were compared using the independent student t-test or the Mann-Whitney U test, as appropriate. Continuous data are expressed as mean and standard deviation (SD) or median (interquartile range [IQR]) values. A *p* < 0.05 was regarded as statistically significant. Covariate imbalance was assessed by calculating standardized differences for the covariates age, gender, coronary artery disease, valvular heart disease, arterial hypertension, diabetes mellitus, atrial fibrillation, atrial flutter, stroke, and heart failure, and also by calculating an omnibus test and significant differences between the two investigated groups using Wilcoxon rank-sum test and χ^2^ test.”

### Patient and Public Involvement

Patients or the public were not involved in the design, or conduct, or reporting, or dissemination of our research.

## Results

With regards to the assessment of covariate imbalance, standardized differences and omnibus test (*p* = 0.556) revealed no statistically significant differences between the two investigated groups (standardized differences >0.25 were considered significant covariate imbalance) (Supplementary Table 6).

The baseline characteristics of both patient cohorts are presented in Table 1. According to matching criteria, the same rates of heart failure, coronary artery disease, and paroxysmal AF were present in both groups at inclusion. Similarly, no significant differences were observed with regards to other comorbidities and predisposing risk factors for cardiac arrhythmias including arterial hypertension, diabetes mellitus and relevant valvular heart disease as well as sex and gender. No significant differences with regards to antiarrhythmics were observed (Table 1). Origin of pneumonia in the control group is depicted in Supplementary Table 4.

**Table 1:** Baseline characteristics.

	**COVID-19 (*n* = 60)**		**Non-COVID-19 (*n*= 60)**		***p***
	***n***	**Mean ± SD, median (Q3–Q1) or %**	***n***	**Mean ± SD, median (Q3–Q1) or %**	
Gender (female)	14/60	23.3%	14/60	23.3%	>0.999
Age (years)	60	66.5 ± 12.6	60	65.9 ± 11.61	0.813
BMI (kg/m^2^)	51	27.7 (5.1)	50	25.6 (6.7)	0.493
**Medical history**					
Arterial hypertension	31/60	51.7%	33/60	55%	0.714
Coronary artery disease	9/60	15.0%	9/60	15.0%	>0.999
Peripheral vascular disease	4/60	6.7%	2/60	3.3%	0.679
Diabetes mellitus	13/60	21.7%	14/60	23.3%	0.827
Current smoking	10/60	16.7%	16/60	26.7%	0.184
Heart failure	7/60	11.7%	7/60	11.7%	>0.999
Valvular heart disease	3/60	5.0%	5/60	8.3%	0.717
Paroxysmal AF	9/60	15.0%	9/60	15.0%	>0.999
Atrial flutter	1/60	1.7%	0/60	0%	>0.999
Pulmonary arterial hypertension	2/60	3.3%	1/60	1.7%	>0.999
Obstructive lung disease	8/60	13.3%	12/60	20.0%	0.327
Structural lung disease	0/60	0%	1/60	1.7%	>0.999
Stroke/TIA	6/60	10.0%	3/60	5.0%	0.491
**Medication**					
Beta-blockers	18/60	30.0%	22/60	36.7%	0.439
NOAK/AOK	7/60	11.7%	8/60	13.3%	0.783
Amiodarone	0/60	0%	2/60	3.3%	0.496

*AF, atrial fibrillation; BMI, body mass index; SD, standard deviation.*

*p <0.05.

**Figure 1:**
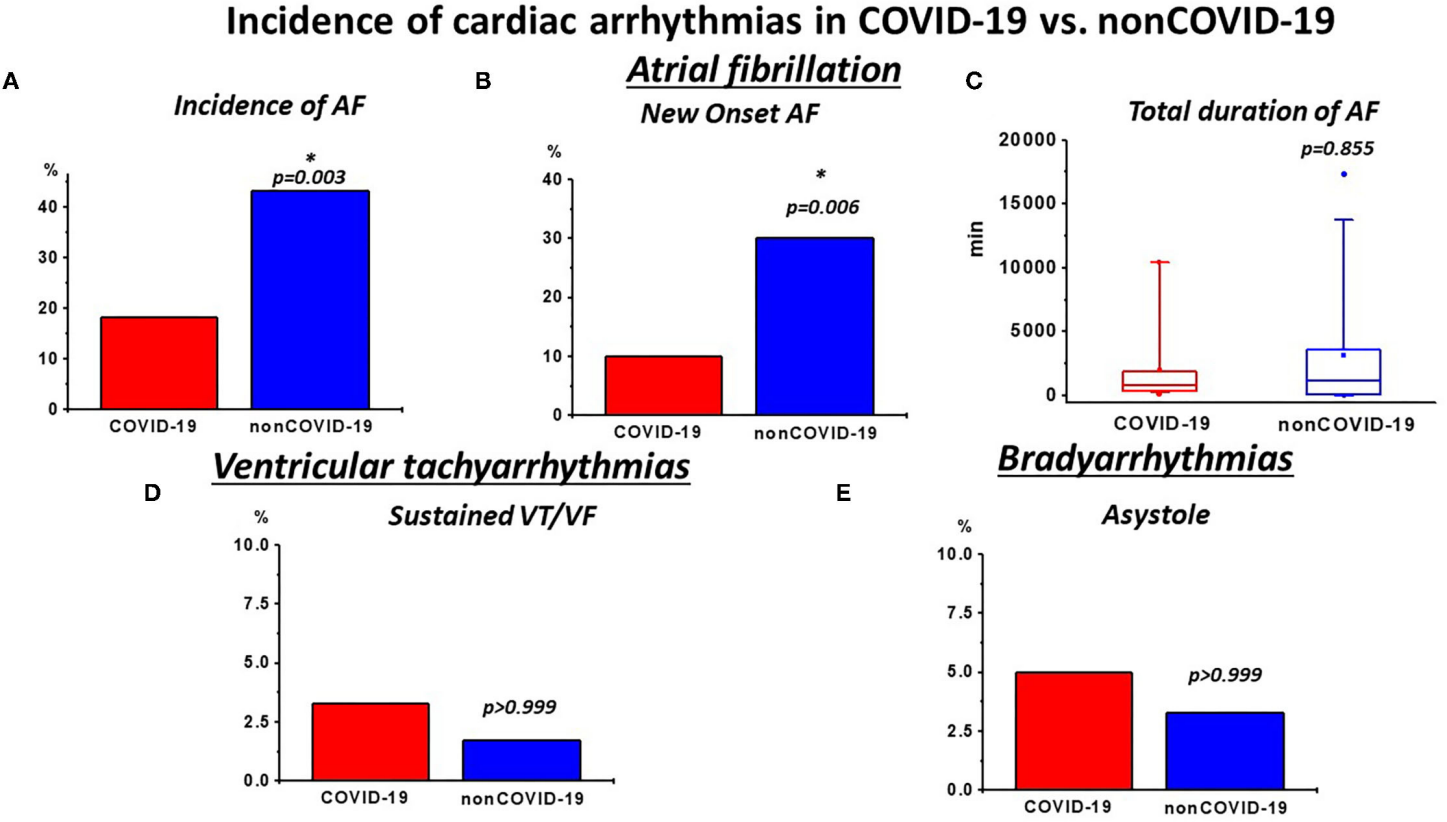
Incidence of relevant cardiac arrhythmias during intensive care (ICU) rhythm monitoring in COVID-19 vs. non-COVID-19: **(A)** incidence of atrial fibrillation (AF) and **(B)** newly diagnosed AF was high in both groups. However, non-COVID-19 patients presented a higher burden of AF and newly diagnosed AF, **(C)** while the total duration of AF was not different in affected patients. **(D)** The incidence of sustained VTs/ventricular fibrillation (VF) was similar in both groups **(E)** and the frequency of asystole was also not significantly different. *p < 0.050.

The analyses of the continuous rhythm monitoring during the ICU stay are presented in Table 2 and Figure 1. Additionally, a separate analysis of patients displaying a QTc-time over 500 ms in the admission ECG is depicted in Supplementary Table 5. Expectedly, COVID-19 presented a high rate of cardiac arrhythmias. Nevertheless, when matched to non-COVID-19, rates of relevant ventricular tachyarrhythmias were similar (Table 1; Figure 1D). With regards to bradyarrhythmias, there was no significant difference in the incidence of high grade AVBs or asystole (Table 1; Figure 1E). Although the rates of AF diagnosed by 12-lead ECG at admission were similar in both groups (Supplementary Table 1), the incidence of AF during rhythm-monitoring was significantly higher in the non-COVID-19 population despite comparable risk factors for the development of arrhythmias. This was reflected by higher rates of AF during ICU stay, but similar AF duration during the monitoring period in affected patients was observed. The higher rates of AF also corresponded to a significantly higher necessity for electrical cardioversion in the non-COVID-19 group (Table 2). Furthermore, the incidence of newly diagnosed AF was significantly higher in non-COVID-19 indicating a more pronounced arrhythmic substrate in this population.

**Table 2:** Continuous rhythm monitoring during ICU stay.

	**COVID-19 (*n* = 60)**		**Non-COVID-19 (*n* = 60)**		***p***
	***n***	**Median (Q3–Q1) or %**	***n***	**Median (Q3–Q1) or %**	
**Supraventricular tachyarrhythmias**					
AF during ICU stay	11/60	18.3%	26/60	43.3%	0.003^*^
New-onset of AF	6/60	10.0%	18/60	30.0%	0.006^*^
Duration of total AF burden (minutes)	60	780.0 (1,680.0)	60	960.0 (4,035.0)	0.855
Other SVTs^$^	5/60	8.3%	8/60	13.3%	0.378
**Ventricular tachyarrhythmias**					
nsVT	4/60	6.7%	4/60	6.7%	>0.999
Sustained VT or VF	2/60	3.3%	1/60	1.7%	>0.999
**Bradyarrhythmias**					
High grade AVB^§^	0/60	0%	0/60	0%	>0.999
Asystole	3/60	5.0%	2/60	3.3%	>0.999
Bradyarrhytmia absoluta	0/60	0%	1/60	1.7%	>0.999
**eCV**	4/60	6.7%	12/60	20.0%	0.029*
**Reason for eCV**					
AF	3/60	5.0%	10/60	16.6%	0.040^*^
Other SVTs^$^	0/60	0%	1/60	1.7%	>0.999
Sustained VT or VF	1/60	1.7%	1/60	1.7%	>0.999

*AF, atrial fibrillation; AVB, atrioventricular block; ICU, intensive care unit; eCV, electrical cardioversion; nsVT, non-sustained ventricular tachycardia; SVT, supraventricular tachycardia; VF, ventricular fibrillation; VT, ventricular tachycardia;*

$ *definition other SVT see method section;*

§ *For definition of high grade AVB see Method section;*

* *p < 0.05*.

With regards to inflammatory activity and disease severity, non-COVID-19 revealed higher leucocytes and procalcitonin (PCT) levels. This was further accompanied by increased lactate levels and decreased pH (**Table 4**) in non-COVID-19. Consequently, while mortality was high in both groups, a significantly higher rate in the non-COVID-19 group was observed (Table 3), indicating a more pronounced critical patient status.

**Table 3:** Patients' outcome and relevant therapies during ICU stay.

	**COVID-19 (*n* = 60)**		**Non-COVID-19 (*n* = 60)**		***p***
	***n***	**Median (Q3–Q1) or %**	***n***	**Median (Q3–Q1) or %**	
**Outcome ICU**					
Death	21/60	35.0%	34/60	56.7%	0.017*
Discharged from ICU	39/60	65.0%	26/60	43.3%	0.017^*^
Duration of ICU stay (days)	60	13.0 (18.0)	60	11.5 (17.0)	0.308
**Required ICU therapy**					
ECMO	9/60	15.0%	14/60	23.3%	0.246
Hemofiltration	17/60	28.3%	25/60	41.7%	0.126
Catecholamines	45/60	75.0%	53/60	88.3%	0.059
Required catecholamines	60	1.0 (1.0)	60	1.0 (1.0)	0.640
**Ventilation therapy**					
NIV	9/60	15.0%	7/60	11.7%	0.591
Intubation	51/60	85.0%	53/60	88.3%	0.591
Duration of intubation	60/60	9.0 (20.0)	60/60	5.0 (10.0)	0.711
Relevant bleedings	4/60	6.7%	4/60	6.7%	>0.999
CPR	4/60	6.7%	6/60	10.0%	0.509
**Reason for CPR**					
Asystole	1/60	1.7%	3/60	5.0%	0.619
VF/VT	1/60	1.7%	1/60	1.7%	>0.999
Pulseless electrical activity	2/60	3.3%	2/60	3.3%	>0.999
Therapeutic anticoagulation	24/60	40.0%	30/60	50.0%	0.271
**Thrombosis/thromboembolic events**					
Pulmonary embolism	10/60	16.7%	2/60	3.3%	0.015^*^
Peripheral thrombosis/thromboembolism	3/60	5.0%	5/60	8.3%	0.717
Stroke/TIA	4/60	6.7%	0/60	0%	0.042^*^

*CPR, cardiopulmonary resuscitation; ECMO, extracorporal membrane oxygenation; ICU, intensive care unit; NIV, non-invasive ventilation; TIA, transient ischemic attack; VF, ventricular fibrillation; VT, ventricular tachycardia.*

* *p < 0.05*.

**Figure 2:**
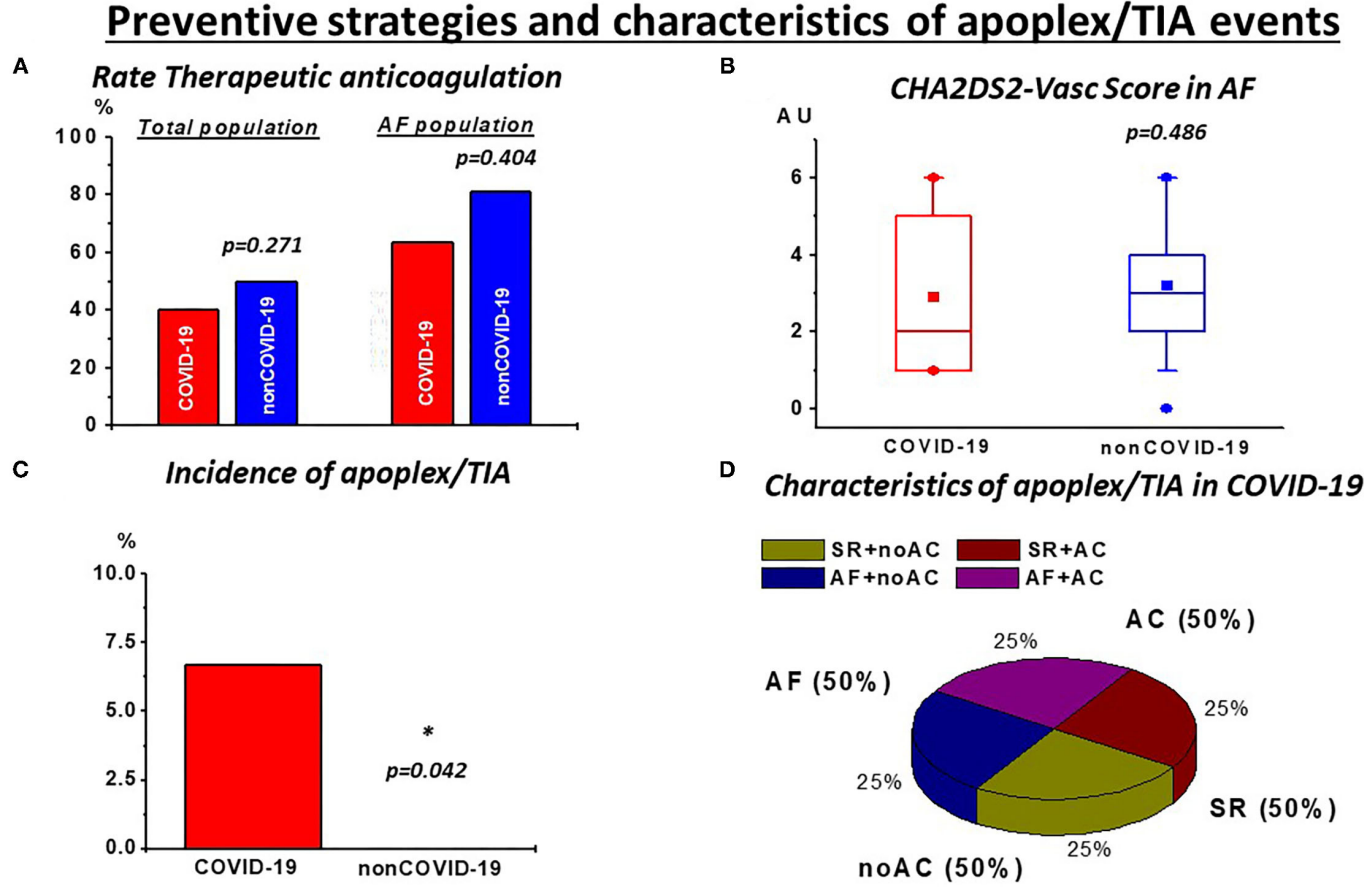
Preventive strategies and characteristics of stroke/TIA events: **(A)** despite high but not different rates of therapeutic anticoagulation (TAC) in the total population and in patients with atrial fibrillation (AF) during rhythm monitoring as well as **(B)** similar CHA2DS2-Vasc Scores in this subgroup, **(C)** incidence of stroke/TIA events was significantly higher in COVID-19. **(D)** These events were also observed with AC (50%) and with continuous sinus rhythm (SR; 50%) during rhythm monitoring. noAC, no application of TAC. *p < 0.05.

In contrast to these observations and in line with previous reports (9), we observed a higher rate of pulmonary embolisms in COVID-19 (Table 3). This observation was consistent with high stroke rates in COVID-19. Of note, despite a lower burden of AF as well as similarly high rates of anticoagulation and comparable CHA_2_DS_2_-Vasc scores, a significantly higher incidence of thrombotic strokes/TIA was revealed (Table 3; Figure 2). Of note, these events were also observed in patients receiving TAC and with continuous sinus rhythm (Figure 2; Supplementary Table 2), indicating disease-specific events that occur independently of cardiac arrhythmias.

## Discussion

The typical finding in severe COVID-19 disease is pneumonia accompanied by acute lung injury (10). In this context, many recent studies covered the topic of COVID-19-related cardiac injury (11, 12). Nevertheless, despite described cases of COVID-19 specific myocarditis, recent publications in critically ill patients, indicated that in this population cardiac injury is rather explained by the high inflammatory burden, similar to cardiac injury in other severe inflammatory processes such as in acute respiratory distress syndrome and severe pneumonia (11, 12). A further point of interest is the burden of arrhythmias in COVID-19 patients. Only a few studies on this topic have been published, pointing toward a high arrhythmic burden in this patient collective (13, 14). Nevertheless, comparable with findings on myocardial injury, arrhythmias and especially AF are a frequent finding in patients with severe pneumonia and sepsis (15). Accordingly, the present study aimed to further evaluate this issue.

To account for underlying medical conditions predisposing to cardiac arrhythmias, the study cohorts were matched for preexisting AF as well as age, gender, coronary artery disease, valvular heart disease, arterial hypertension, diabetes mellitus, atrial fibrillation, atrial flutter, stroke, and heart failure. Reliability of the matching process was further confirmed by analyzing covariate imbalance between the two investigated, showing no significant differences.

In contrast to former studies conducted on arrhythmias in COVID-19, the present project included monitoring data on arrhythmias for the entire ICU-stay in addition to standard 12-lead ECGs, thus allowing for a more precise analysis of the arrhythmic burden. To avoid potential interference of novel treatment options, such as dexamethasone, with our findings, patients were recruited during the first wave of the pandemic before publication of the “RECOVERY Trial in July 2020.” The impact of COVID-19 disease on myocardial arrhythmias might be better reflected through this approach, since outcomes are not influenced by this treatment regime, which is now routinely applied in the involved study centers. Accordingly, COVID-19 specific therapy is low in the present patient collective as it was mostly experimental during this investigated period.

In the COVID-19 group, AF was the most frequent arrhythmia and was observed in 18.3% of all patients (Table 2; Figure 1A). It is in line with a recent publication by Bertini et al. (14), which reported an AF rate of about 22% in a similar patient collective, documented by ECG at hospital admission. Of note, the mean patient age in that study tended to be higher compared with our collectives, which might explain the slightly higher AF rate (14). Interestingly, the AF burden in the non-COVID-19 group in our study was even higher, ranging at around 43% and requiring a higher need for electrical cardioversion (Table 2; Figure 1A). This was also reflected by an increased incidence of new-onset of AF (Table 2; Figure 1B), indicating a more pronounced proarrhythmic substrate in this population. Of note, the rates of AF in our control group are in line with former studies conducted on AF in sepsis and septic shock, with rates of new onset of AF ranging between 7% and 46%, depending on disease severity (16, 17). Apart from AF, rates of other supraventricular and also ventricular tachyarrhythmias and relevant bradyarrhythmias with consequent need for cardiopulmonary resuscitation were similar in both COVID-19 and non-COVID-19 patients (Tables 2, 3; Figures 1D,E). With respect to ventricular arrhythmias, one has to keep in mind the comparably low amount of heart failure (11.7%) and coronary artery disease (15%) in our patient collective resulting in a low percentage of patients with a predisposing myocardial substrate, which could facilitate ventricular tachycardias (VT).

Since inflammatory processes are known to increase the vulnerability for arrhythmias (15, 18), the higher inflammatory burden and disease severity in the non-COVID-19 group, reflected by higher levels of leucocytes, PCT, lactate, and also lower pH levels with consequent higher mortality rates in non-COVID-19 (Tables 3, 4), represents an important factor in this regard (15). Consequently, one might speculate that similar to other critically ill patients, in COVID-19, cardiac arrhythmias are primarily driven by inflammatory processes and disease burden, rather than by disease-specific effects of COVID-19.

**Table 4:** Relevant laboratory markers during ICU stay.

	**COVID-19 (*n* = 60)**		**Non-COVID-19 ((*n* = 60)**		***p***
	***n***	**Median (Q3–Q1)**	***n***	**Median (Q3–Q1)**	
Lactate (U/L)	60	2.6 (2.1)	60	3.5 (4.8)	0.017^*^
Min. pH	60	7.19 (0.1)	60	7.13 (0.1)	0.045^*^
Creatinine (mg/dl)	60	1.7 (2.1)	60	2.3 (2.6)	0.404
Min potassium (mmol/L)	60	3.4 (0.4)	60	3.3 (0.5)	0.720
Leukocytes (10^9^/L)	60	14.8 (11.5)	60	20.2 (11.8)	0.002^*^
Min. lymphocytes (10^9^/L)	60	4.4 (6.6)	45	4.9 (6.5)	0.712
CRP (ng/ml)	59	25.5 (17.7)	60	28.2 (15.4)	0.493
PCT (ng/ml)	60	1.9 (5.1)	57	3.0 (17.9)	0.013^*^
Interleukin 6 (pg/ml)	53	513.8 (2,395.2)	23	394.8 (1,080.6)	0.923
Fibrinogen (mg/dl)	34	672.5 (298)	54	602.5 (270.0)	0.175

*CRP, C-reactive protein; Min., lowest level of laboratory biomarker obtained during the total period of ICU stay; PCT, procalcitonin. Relevant laboratory findings obtained during intensive care unit (ICU) stay. If not other indicated, the highest obtained value during the whole period of ICU stay is presented.*

* *p < 0.05*.

While no significant increase of arrhythmias in the COVID-19 cohort was evident, thromboembolic events showed a significant increase compared with non-COVID-19 patients. This is reflected by a higher incidence of pulmonary embolism and stroke/TIA in our COVID-19 cohort (Table 3). Accordingly, this finding suggests a COVID-19 specific thromboembolic effect independent of arrhythmic burden. In AF, the most common observed arrhythmia in our study, TAC, is recommended according to preexisting risk factors with a high risk of thromboembolic strokes (6). This therapy is known to be efficient as indicated in our non-COVID-19 cohort with no stroke stoke/TIA events despite a higher incidence of AF (Table 2) but also with a high rate of TAC (Figure 2A). Whether in COVID-19, AF and the associated preexisting risk factors might further drive thromboembolic events, is still a matter of debate. Concerning our results, this seems questionable since the rate of neurologic events was higher in COVID-19 despite a lower incidence of AF, comparable CHA2DS2-Vasc scores (Figures 2B,C) and also the appearance of these events in patients with continuous sinus rhythm during monitoring (Figure 2D). This emphasizes the need for effective prevention strategies in critically ill COVID-19. However, in our study, the rate of TAC in critically COVID-19 was comparable with non-COVID-19, despite the lower rate of AF (Figure 2A). It could be argued, that, given the high incidence of thromboembolic events, more, if not all critical COVID-19 patients should receive effective anticoagulation. While a mortality benefit seems to be associated with anticoagulatory treatment in COVID-19, the clinical evidence for efficacy and safety of such an approach is a topic for ongoing investigations (19, 20). Importantly, we observed thrombotic/thromboembolic neurological events despite sufficient TAC (Figure 2D; Supplementary Table 2), indicating TAC to be probably less effective in this population. Thus, taken together our data emphasize that thromboembolic events seem to be a disease-specific in severe COVID-19 patients unrelated to the presence of arrhythmias.

## Limitations

The present study has by design its limitations, while contributing novel clinical findings. Our sample size may be too small to detect differences in arrhythmias with low incidence, such as ventricular tachyarrhythmias and bradyarrhythmias. The heterogeneity of our comparison group, which consists of patients suffering from pneumonia of diverse origin might in part differ with regards to the pathogenetic mechanisms compared with COVID-19 pneumonia. Thus, the findings of the present study have to be considered as hypothesis generating. The passionate use of untested treatments in a number of COVID-19 patients (such as tocilizumab or hydroxychloroquine, Supplementary Table 3) might have affected the results, especially concerning arrhythmia burden due to effects on QT interval. However, while QT prolongation is suspected to promote this issue, QTc in our COVID-19 cohort was in the normal range with shorter QTc compared with non-COVID-19 (Supplementary Table 1). Instead of screening, diagnostic workups for thromboembolic events were only performed when clinically suspected and therefore, they are probably underestimated.

In summary, AF is common in severe COVID-19, but we found it to be less frequent than in severe pneumonia of non-COVID-19 origin. Arrhythmia might be mainly attributed to a high inflammatory activity and disease severity, instead of a COVID-19 specific mechanism. The contrasting higher incidence of stroke, despite the lower rate of AF, seems to be a disease-specific feature of critical COVID-19, consistent with high rates of pulmonary embolisms. Further research will hopefully clarify the potential role of TAC to prevent thromboembolic events, which are independent of AF.

## Data Availability Statement

The raw data supporting the conclusions of this article will be made available by the authors, without undue reservation.

## Ethics Statement

The studies involving human participants were reviewed and approved by University Hospital Münster: Nr. 2020-306-f-S, Maria Hilf Hospital Mönchengladbach: Nr. 143/2020, and University Hospital Salzburg: Nr. 1071/2020. Written informed consent for participation was not required for this study in accordance with the national legislation and the institutional requirements.

## Author Contributions

All authors listed have made a substantial, direct, and intellectual contribution to the work and approved it for publication.

## Conflict of Interest

The authors declare that the research was conducted in the absence of any commercial or financial relationships that could be construed as a potential conflict of interest.

## Publisher's Note

All claims expressed in this article are solely those of the authors and do not necessarily represent those of their affiliated organizations, or those of the publisher, the editors and the reviewers. Any product that may be evaluated in this article, or claim that may be made by its manufacturer, is not guaranteed or endorsed by the publisher.
