# Supplementary material for: Higher Incidence of Stroke in Severe COVID-19 Is Not Associated With a Higher Burden of Arrhythmias: Comparison With Other Types of Severe Pneumonia
Source: Front Cardiovasc Med. 2021 Nov 24;8:763827. doi: 10.3389/fcvm.2021.763827 (PMC8652060; doi:10.3389/fcvm.2021.763827)
Supplement: Supplementary file 1 [file Table_1.DOCX]

**Supplemental methods:**

Substances used for therapeutic anticoagulation in our patient cohort comprise Dalteparin (200 IE/kg o.d.) and Enoxaparin (1 mg/kg b.i.d.) adjusted to bodyweight. In the presence of acute and/or chronic renal failure, dose was adjusted according to Anti-Faktor Xa analysis. As alternative, unfractionated heparin, adjusted to PTT was used in the presence of renal failure. Therapeutic anticoagulation was started in patients with atrial fibrillation with CHA2DS2-VASc-Score ≥ 1 as well as in patients with thromboembolic events. Therapeutic anticoagulation was also started in COVID-19 patients after careful clinical evaluation of the thrombotic/thromboembolic risk, according to physician guided decision making. All remaining patients received prophylactic anticoagulation with Dalteparin (5000IE o.d.) or Enoxaparin (40mg o.d.) – half the dosage was applied in case of renal failure for prophylactic anticoagulation.

**Supplemental table S1 Twelve lead ECG at admission on ICU**

|  | **COVID-19 (n=29)** | | **NonCOVID-19 (n=54)** | |  |
| --- | --- | --- | --- | --- | --- |
|  | **n** | **Median(Q3-Q1) or %** | **n** | **Median(Q3-Q1) or %** | ***p-*value** |
| Heart rate | 29 | 85.5(39.0) | 54 | 92.0(30.0) | 0.422 |
| **Cardiac Rhythm**  Sinus rhythm  Atrial fibrillation  Atrial flutter  Atrial pacemaker | 25/29  4/29  0/29  0/29 | 86.2%  13.8%  0%  0% | 47/54  5/54  1/54  1/54 | 87.0%  9.3%  1.9%  1.9% | >0.999  0.713  >0.999  >0.999 |
| Axis deviation  Normal axis  Left axis  Right axis | 10/29  18/29  1/29 | 35.5%  62.1%  3.4% | 24/54  28/54  2/54 | 44.4%  51.9%  3.7% | 0.379  0.372  >0.999 |
| S1Q3-type | 3/29 | 10.3% | 4/54 | 7.4% | 0.691 |
| Left Bundle branch block | 0/29 | 0% | 3/54 | 5.6% | 0.310 |
| Right bundle branch block | 1/29 | 3.4% | 4/54 | 7.4% | 0.653 |
| PQ (ms) | 25 | 150.0(25.0) | 48 | 150.0(46.0) | 0.312 |
| QRS (ms) | 29 | 90.0(18.0) | 54 | 90.0(20.0) | 0.874 |
| QTc (ms) | 29 | 440.0(30.0)  min. 378  max. 500 | 54 | 452.0(38.0)  min. 400  max. 590 | 0.018* |
| Disturbance of repolarization^$^ | 9/29 | 31.0% | 10/54 | 18.5% | 0.273 |

ICU, intensive care unit; ^$^Disturbances of repolarization was defined as at least ST-elevation, ST-depression and/or negative T-waves in two of the recorded leads; **p*< 0.05.

**Supplemental table S2 Characteristics of COVID-19 patients suffering from TIA/Stroke**

|  | **1** | **2*** | **3** | **4** |
| --- | --- | --- | --- | --- |
| Sex | male | female | male | male |
| Age (years) | 77 | 89 | 49 | 60 |
| History of congestive Heart Failure | no | no | no | no |
| History of arterial hypertension | yes | no | no | no |
| History of diabetes Mellitus | no | no | no | no |
| History of Stroke/TIA/Thromboembolism | no | yes | no | no |
| Vascular disease | yes | no | no | no |
| CHA_2_DS_2_-VASc-Score | 4 | 5 | 0 | 0 |
| History of atrial fibrillation | yes | no | no | no |
| History of atrial flutter | no | no | no | no |
| Aftrial Fibrillation during ICU stay | yes | yes | no | no |
| Duration of Atrial fibrillation during ICU stay (min.) | 4080 | 180 | 0 | 0 |
| VT/VF during ICU stay | no | no | no | yes |
| Electric Cardioversion during ICU stay | no | no | no | yes |
| Pulmonary embolism during ICU stay | no | no | no | yes |
| Peripheral thrombosis during ICU stay | no | no | no | yes |
| Therapeutic AC before admission | NOAC | no | no | no |
| Therapeutic AC during ICU stay | LMWH | no | no | LMWH |
| Died on ICU | yes | yes | yes | no |

AC, anticoagulation; ICU, intensive care unit; LMWH=low molecular weight heparin; NOAC, novel oral anticoagulants; VF, ventricular fibrillation; TIA, transient ischemic attack; VT, ventricular tachycardia; *Anticoagulation therapy was never initiated due to early death at ICU.

**Supplemental table S3 Specific therapies aiming COVID-19 in COVID-19 patients**

|  | **COVID-19 (n=60)** | |
| --- | --- | --- |
|  | **n** | **%** |
| **Hydroxychloroquine** | 11/60 | 18.3% |
| **Remdesivir** | 1/60 | 1.7% |
| **Taclizumab** | 12/60 | 20.0% |
| **Sarilumab** | 1/60 | 1.7% |
| **Convalescent plasma** | 2/60 | 3.3% |

**Supplemental table S4 Origin of pneumonia in nonCOVID-19 patients**

| **non-COVID – n = 60** | | |
| --- | --- | --- |
| **Origin of pneumonia n** | | |
| Viral 19 | | |
| Bacterial 37 | | |
| Toxic 4 | | |
| **Pathogen** | **n** | **n** |
| **Bacteria**  **gram-positive** | **primary infection** | **secondary infection** |
| Staphylococcus | 4 | 3 |
| Streptococcus | 7 | 0 |
| Aktinomyces | 0 | 1 |
| Corynebacterium | 1 | 0 |
|  |  |  |
| **Bacteria**  **gram-negative** | **primary infection** | **secondary infection** |
| Klebsiella | 2 | 0 |
| Escherichia | 2 | 0 |
| Haemophilus | 0 | 1 |
| Pseudomonas | 1 | 0 |
| Chlamydia | 1 | 1 |
|  |  |  |
|  |  |  |
| **dsDNA virus** | **primary infection** | **secondary infection** |
| Herpes Simplex | 0 | 3 |
| Epstein-Barr virus | 0 | 1 |
|  |  |  |
| **ssRNA virus** | **primary infection** | **secondary infection** |
| Influenza | 14 | 0 |
| Respiratory syncitial virus | 1 | 0 |

| **Patient** | **1** | **2** | **3** | **4** | **5** | **6** | **7** | **8** | **9** |
| --- | --- | --- | --- | --- | --- | --- | --- | --- | --- |
| **Covid** | yes | yes | no | no | no | no | no | no | no |
| **Sex** | m | f | f | m | m | f | f | f | m |
| **Age** | 57 | 49 | 88 | 78 | 49 | 80 | 74 | 41 | 60 |
| **VT during ICU stay** | no | no | no | no | no | no | no | no | no |
| **nsVT during ICU stay** | no | no | no | no | no | no | no | no | no |
| **Death on ICU** | no | no | yes | yes | yes | yes | no | yes | no |
| **Pulmonary embolism during ICU stay** | no | no | no | no | no | no | no | no | no |
| **Stroke/TIA during ICU stay** | no | no | no | no | no | no | no | no | no |
| **Therapeutic anticoagulation during ICU stay** | no | no | yes | yes | yes | no | yes | no | no |
| **History of AF** | no | no | yes | yes | no | no | yes | no | no |
| **AF during ICU stay** | no | no | yes | yes | no | no | yes | no | no |
| **New Onset AF during ICU stay** | no | no | no | no | no | no | no | no | no |
| **QRS at ICU admission** | 130 | 80 | 134 | 116 | 86 | 148 | 94 | 160 | 126 |
| **QTc at ICU admission** | 500 | 500 | 536 | 525 | 503 | 509 | 500 | 570 | 590 |
| **Heart rate at ICU admission** | 80 | 126 | 101 | 154 | 72 | 92 | 83 | 82 | 90 |
| **Number of VES on ICU admission ECG** | 0 | 0 | 0 | 0 | 0 | 0 | 1 | 0 | 0 |
| **Number of SVES on ICU admission ECG** | 0 | 0 | 0 | 0 | 0 | 0 | 0 | 0 | 0 |

**Supplemental table S5 Characteristics of patients with a QTc-interval <500ms in the ICU admission ECG**

VT, ventricular tachycardia; nsVT, non-sustained ventricular tachycardia; ICU, intensive care unit; TIA, transient ischemic attack; AF, atrial fibrillation; VES, ventricular extrasystole; SVES, supraventricular extrasystole

| **Covariate** | **Standardized difference** | **p-value** |
| --- | --- | --- |
| **Age** | 0.045 | 0.8132 |
| **Gender** | <0.001 | >0.999 |
| **KHK** | <0.001 | >0.999 |
| **Valvular Heart disease** | 0.134 | 0.714 |
| **Art. Hypertension** | 0.067 | 0.8548 |
| **Diabetes mellitus** | 0.040 | >0.999 |
| **Atrial fibrillation** | <0.001 | >0.999 |
| **Atrial flutter** | 0.216 | 0.9822 |
| **Stroke/TIA** | 0.139 | 0.7897 |
| **Heart Failure** | <0.001 | >0.999 |

**Supplement table S6: Analysis of Covariate imbalance**

Standardized differences and omnibus test (p= 0.556) revealed no statistically significant covariate imbalance between the two investigated groups (standardized differences >0.25 were considered significant covariate imbalance).
