# Supplementary figures and images for: Higher Incidence of Stroke in Severe COVID-19 Is Not Associated With a Higher Burden of Arrhythmias: Comparison With Other Types of Severe Pneumonia
Source: Front Cardiovasc Med. 2021 Nov 24;8:763827. doi: 10.3389/fcvm.2021.763827 (PMC8652060; doi:10.3389/fcvm.2021.763827)

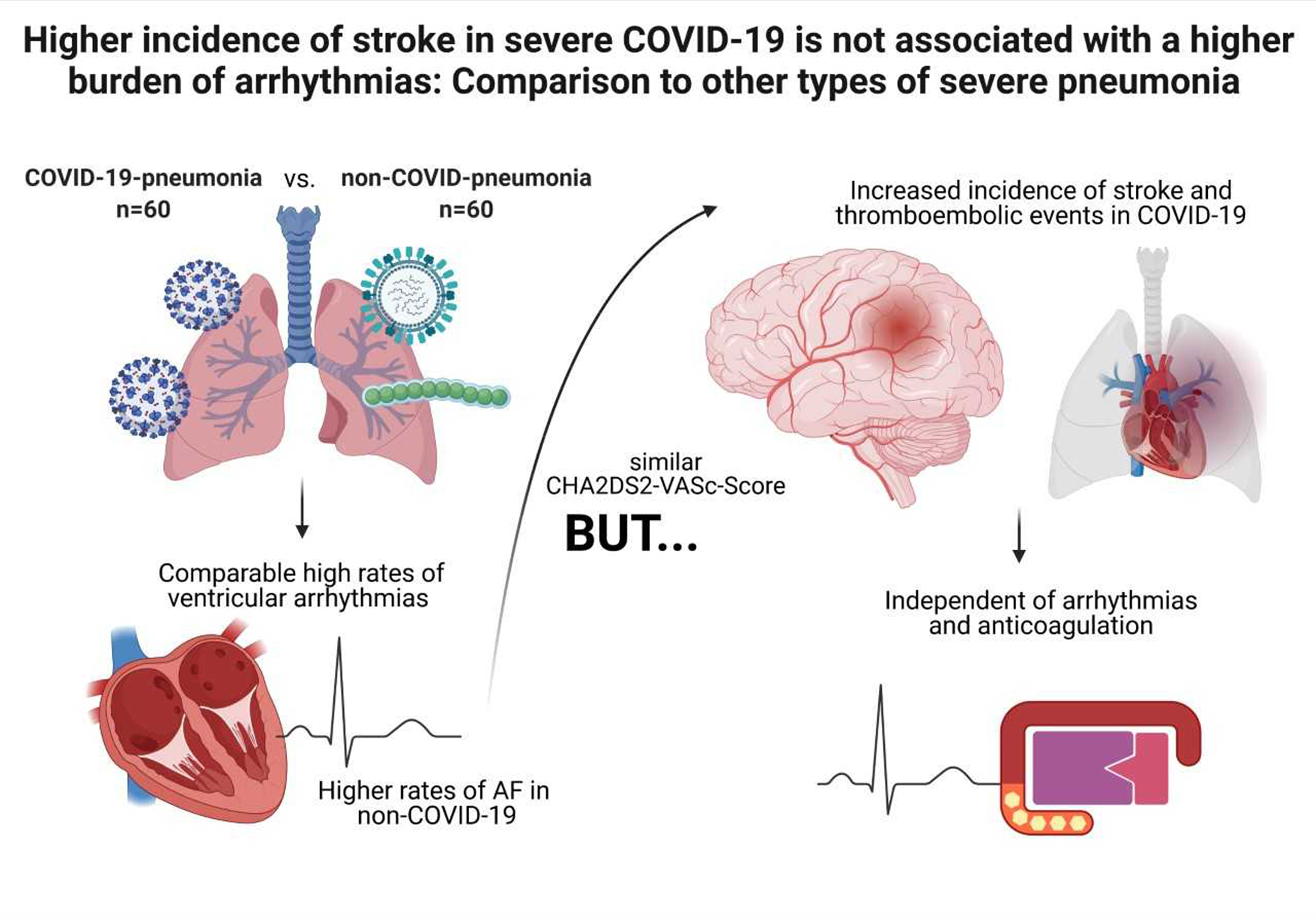

Supplement: Supplementary file 2 [file Image_1.JPEG]
